# Efficient Degradation of Ofloxacin by Magnetic CuFe_2_O_4_ Coupled PMS System: Optimization, Degradation Pathways and Toxicity Evaluation

**DOI:** 10.3390/toxics12100731

**Published:** 2024-10-10

**Authors:** Chuanhong Xing, Kang Chen, Limin Hu, Lanhua Liu

**Affiliations:** School of Ecology and Environment, Zhengzhou University, Zhengzhou 450001, China; chxing@zzu.edu.cn (C.X.); ck915543328@163.com (K.C.)

**Keywords:** CuFe_2_O_4_, peroxymonosulfate, radical oxygen species, response surface methodology, degradation pathways

## Abstract

Magnetic CuFe_2_O_4_ was prepared with the modified sol–gel method and used for enhanced peroxymonosulfate (PMS) activation and ofloxacin (OFL) degradation. The OFL could almost degrade within 30 min at a catalyst dosage of 0.66 g/L, PMS concentration of 0.38 mM, and initial pH of 6.53 without adjustment, using response surface methodology (RSM) with Box-Behnken design (BBD). In the CuFe_2_O_4_/PMS system, the coexisting substances, including CO_3_^2−^, NO_3_^−^, SO_4_^2−^, Cl^−^ and humic acid, have little effect on the OFL degradation. The system also performs well in actual water, such as tap water and surface water (Mei Lake), indicating the excellent anti-interference ability of the system. The cyclic transformation between Cu(II)/Cu(I) and Fe(III)/Fe(II) triggers the generation of active radicals including SO_4_^•−^, •OH, •O_2_^−^ and ^1^O_2_. The OFL degradation pathway, mainly involving the dehydrogenation, deamination, hydroxylation, decarboxylation and carboxylation processes, was proposed using mass spectroscopy. Moreover, the toxicity assessment indicated that the end intermediates are environmentally friendly. This study is about how the CuFe_2_O_4_/PMS system performs well in PMS activation for refractory organic matter removal in wastewater.

## 1. Introduction

With the development of population growth, climate change and intensive exploitation of water resources, freshwater scarcity has become one of the most significant ecological threats [1]. While wastewater recycling assists in fulfilling the increasing demands, emerging pollutants like pharmaceuticals and personal care products (PPCPs) continue to jeopardize the health of humans [2]. Fluoroquinolones (FQs) are a kind of broad spectrum antibiotic that target bacterial deoxyribonucleic acid (DNA). Due to its potent antibacterial activity against both Gram-negative and Gram-positive bacteria, the consumption of ofloxacin (OFL) accounts for one-fifth of the global consumption of FQs in aquaculture, animal husbandry and medical treatment [3,4,5]. However, its negative effects mainly include ecotoxicity, decreased microbial diversity for the ecological environment could not be ignored [6]. Therefore, there is an urgent need to find an efficient strategy to degrade OFL in aquatic environments. 

For OFL degradation, persulfate-based advanced oxidation processes (AOPs) have received much attention for their rapid degradation performance, easy operation and thorough mineralization capability with the generation of a sulfate radical (SO_4_^•−^), •OH and other potent radicals. Compared to the traditional AOPs, persulfate-based AOPs have a series of advantages such as the multiple active species with certain selectivity and long half-life, wide-operating pH range, and ease of storage for persulfate [7]. In comparison to peroxydisulfate (PDS), peroxymonosulfate (PMS) is prone to be activated easily, owing to its asymmetric structure of the OO bond [8]. Currently, the main activation methods can be classified into two categories. One is energy-intensive homogeneous activation systems with high-efficiency activity, but high energy consumption and equipment costs, such as thermal [9], radiation [10], microwave [11] and ultrasonic activation [12]. The other one is heterogeneous activation systems based on transition metal-involved catalysts, with the advantages of simplicity, sustainability and efficiency [13]. Herein, it is crucial to develop an efficient, recyclable and easily manufacturable transition metal-involved catalyst to apply to this technology.

Currently, the most extensively studied transition metal-involved catalysts for PMS activation include Fe [14], Co [15], Cu [16] and Mn [17]. However, single transition metal ions exhibit poor stability and performance in practical applications. Moreover, it is difficult to consider both recovery and catalytic performance for powdered samples. In comparison to monometallic oxide, magnetic spinel materials containing two different metal ions possess the advantage of easy separation and demonstrate excellent stability during PMS activation. This can be attributed to the synergistic redox coupling between the two metal ions [18]. Among these spinel materials, on one hand, bimetallic iron-based catalysts are readily available, non-toxic and exhibit excellent activation performance [19]. Research has shown that the presence of Fe promotes the enrichment of hydroxyl groups on the catalyst’s surface, facilitating the formation of M-OH complexes and as a result, enhancing PMS activation [20]. On the other hand, copper (Cu) exhibits more activation pathways compared to other transition metals, with the generation of a greater number of free radicals and non-radicals (such as SO_4_^•−^, •OH, •O_2_^−^, singlet oxygen, Cu(III)) [21]. Therefore, iron–copper spinel is considered an ideal catalyst for PMS activation, with excellent catalytic performance in organic compound degradation. Due to its low-temperature synthesis and its products of homogeneity and high-purity, the sol–gel combustion method [22] is widely used for iron–copper spinel preparation. 

In this study, an improved sol–gel combustion method was employed to synthesize iron–copper spinel nanoparticles, which showed efficient PMS activation to remove OFL in water. First, the physicochemical properties of CuFe_2_O_4_ were characterized in detail. Second, the optimal reaction condition was evaluated with the help of the Box-Behnken design (BBD) with response surface methodology (RSM). Third, the mechanisms of PMS activation were studied, including the changes in the element valence states and oxygen species of the sample before and after reaction by XPS, the generation of active species by the quenching experiment and electron spin resonance (ESR). Fourth, with the determination of possible intermediates, the possible degradation pathway was clarified for OFL removal in the CuFe_2_O_4_/PMS system. In addition, the toxicities of intermediates were evaluated with quantitative structure–activity relationship predictions.

## 2. Materials and Methods

### 2.1. Chemicals and Reagents

Text S1 contains a complete list of all chemicals and reagents in this research.

### 2.2. Preparation of CuFe_2_O_4_

The CuFe_2_O_4_ was prepared using a modified sol–gel combustion method [23]. First, 2.42 g of Cu(NO_3_)_2_·3H_2_O and 8.06 g of Fe(NO_3_)_3_·9H_2_O (with a molar ratio of 1:2) were dissolved in 50 mL of ultrapure water. The mixture was stirred under a magnetic stirrer at room temperature until the regents dissolved. Second, 5.76 g of citric acid was added to the mixed solution at 60 °C for a certain time (20 min or 2 h) under a water bath. Third, the homogeneous solution obtained was transferred to a drying oven at 125 °C for a certain time (5 h, 7 h and 9 h) to obtain the precursor material. Fourth, the precursor was ground and calcined at 400 °C for 2 h in a muffle furnace with a programming temperature of 90 min. After cooling to room temperature, the prepared catalyst was obtained and labelled as CFO_(x)(y)_ (x = 20 min or 2 h) (y = 5 h, 7 h or 9 h), where x represented the water bath heating time and y represented the drying time.

### 2.3. Characterizations of CuFe_2_O_4_

The relevant characterization approaches are described in Text S2.

### 2.4. Evaluation of Catalytic Performance

The detailed degradation procedure is characterized in Text S3.

## 3. Results and Discussion

### 3.1. Characterization

**Figure 1:**
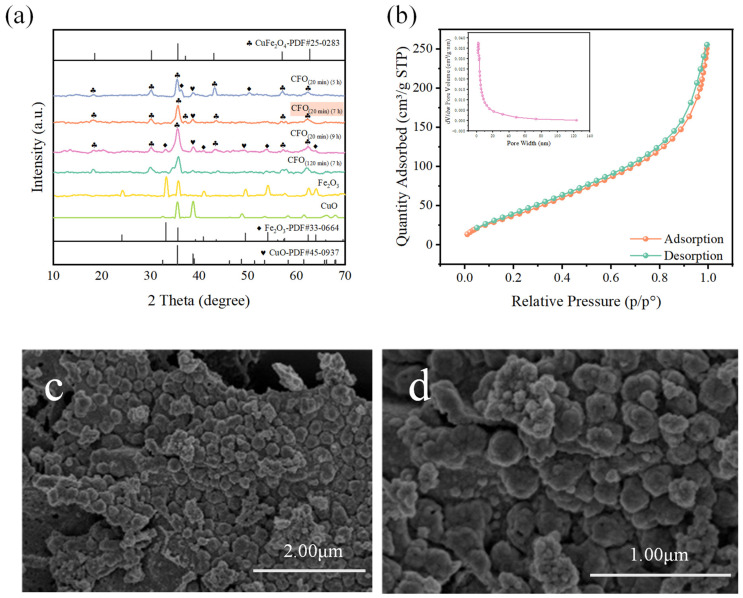
(**a**) XRD patterns for various samples, (**b**) pore size distribution and N_2_ adsorption–desorption isotherms of CuFe_2_O_4_, (**c**,**d**) SEM images of CuFe_2_O_4_.

The XRD spectra of samples with different preparation conditions (focus on water bath heating time and drying time) are shown in Figure 1a. In addition, the corresponding samples for PMS activation are also shown in Figure S1. Under the same water bath heating times (20 min) during sample preparation, the drying time influenced the crystal structure of samples. 2θ at 18.2°, 30.1°, 35.4°, 37.1°, 43.1°, 57.0° and 62.6° correspond to the (111), (220), (311), (222), (400), (511) and (440) facets of CuFe_2_O_4_ (standard card PDF #25-0283) when the drying time was 7 h. There was also a less pronounced diffraction peak at 39.0°, corresponding to (200) facet of CuO (standard card PDF #45-0937). When the drying time was too short (5 h) or too long (9 h), in addition to the above diffraction peaks, diffraction peaks of Fe_2_O_3_ were also observed. Moreover, the different drying times of samples performed different catalytic activity with 71.04%, 95.65% and 81.90% when the drying times were 5 h, 7 h and 9 h respectively. This indicates that 7 h is the optimal drying time for CuFe_2_O_4_ preparation. Then, the water bath heating time was also considered compared to the referring papers [23]. The XRD spectrum of the sample with 20 min water bath heating treatment was similar to that of the samples with 120 min treatment, while the degradation efficiency of OFL was slightly decreased by 3.87% when the water bath heating time was increased from 20 min to 120 min. Herein, the water bath heating time of 20 min and drying time of 7 h might be the optimal preparation conditions for CuFe_2_O_4_, and the characteristics of CuFe_2_O_4_ prepared by this condition will be further analyzed in the following research. 

In addition, monometallic oxides are obtained by the same preparation method, but with the addition of only one type of metal salt. Their corresponding diffraction peaks match those of Fe_2_O_3_ (PDF #33-0664) and CuO (PDF #45-0937), respectively. In terms of their catalytic activity, only 35.31% of OFL was degraded under the same reaction conditions, which demonstrated that iron has a limited ability to activate PMS. Even though the system with CuO has a similar degradation efficiency (89.83%) to OFL, the lack of magnetism limits its recovery from solution. All in all, the bimetallic oxides of CuFe_2_O_4_ that can be selected, not only possess the outstanding PMS activation ability, but also easily recover from solution.

Figure 1b presents the N_2_ adsorption–desorption isotherms and pore size distribution of CuFe_2_O_4_. Its adsorption isotherm belongs to Type II, characterized by an H_3_ hysteresis loop, indicating non-restricted monolayer–multilayer adsorption. The sample consists of particles with slit-like pores [24]. It can be observed that CuFe_2_O_4_ predominantly possesses mesopores, and the capillary condensation phenomenon in the adsorption isotherm indicates the presence of micropores and narrow mesopores. The BET surface area calculated from the linear part of the BET plot (P/P_0_ = 0.29) is 145.2 m^2^/g. According to the desorption branch of the nitrogen isotherm by the BJH method with the Halsey equation, the average pore diameter of CuFe_2_O_4_ is 7.3381 nm. The total pore volume calculated from the volume of N_2_ adsorbed at P/P_0_ = 0.990 is 0.369 cm^3^/g. This result indicates that CuFe_2_O_4_ exhibits a relatively high specific surface area, which is beneficial for catalytic activity with numerous active sites.

The SEM images of representative CuFe_2_O_4_ are shown in Figure 1c. They reveal the dense array of the catalyst particles with a diameter of approximately 200 nm. In addition, the uneven surface of nanoparticles means large interfaces that could serve as abundant active sites for the activation of PMS. The EIS Nyquist curves of CuFe_2_O_4_, CuO and Fe_2_O_3_ are displayed in Figure S2. It is well known that smaller arc radii indicate lower charge transfer impedance. It is evident that CuFe_2_O_4_ has a lower impedance compared to CuO and Fe_2_O_3_, indicating its superior electron transfer ability, which is advantageous for PMS activation.

### 3.2. Catalytic Performance of CuFe_2_O_4_/PMS System in OFL Removal

#### 3.2.1. OFL Degradation in Different Processes

**Figure 2:**
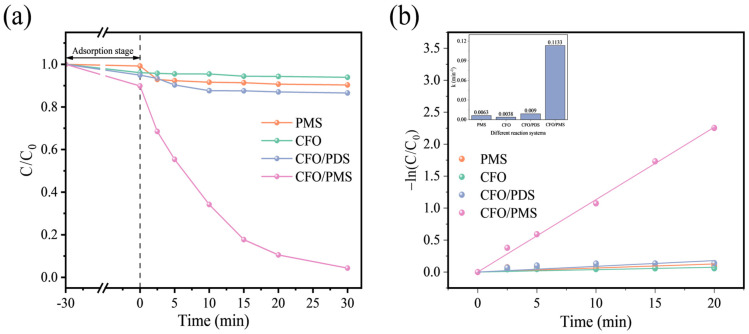
(**a**) Degradation rate and (**b**) corresponding reaction rate constant of OFL in different systems (pH = 6.5 (unadjusted), [OFL]_0_ = 10 mg/L, [CuFe_2_O_4_]_0_ = 0.5 g/L, [PMS]_0_ = 0.4 mM).

In general, the removal of pollutants by heterogeneous catalysis involves physical adsorption and chemical oxidation degradation. Therefore, a 30-min adsorption–desorption equilibrium experiment was conducted to avoid the effect of the adsorption of CuFe_2_O_4_ during the PMS activation process. As shown in Figure 2a, without the addition of PMS, the material exhibited a removal rate of less than 7.00% for OFL, indicating a negligible contribution of adsorption to OFL removal. To quantitatively study the degradation kinetics of OFL, the modified Langmuir–Hinshelwood model was employed as expressed by the following:

(1) $$-{\ln(C/C}_{0})=\mathrm{kt}$$

where C, C_0_, k and t present the concentration of OFL at t, the initial concentration of OFL, the reaction rate constant and the reaction time at t, respectively.

The degradation efficiencies of OFL under different systems are illustrated in Figure 2. When PMS was added alone, OFL was hardly degraded (9.69%), indicating the challenge of PMS activation and oxidation degradation of OFL under ambient conditions. However, with the introduction of CuFe_2_O_4_, the degradation rate of OFL significantly increased (95.65%). This enhancement can be primarily attributed to the efficient activation of PMS by CuFe_2_O_4_. Furthermore, when PMS was replaced by PDS, the degradation efficiency of OFL decreased significantly (13.47%), suggesting that PMS has a more readily activated asymmetric structure compared to PDS in the presence of an iron- and copper-involved system. Additionally, Figure 2b further indicates that the CuFe_2_O_4_/PMS system has the highest degradation reaction rate constant of 0.1133 min^−1^, which is 17.98, 29.82 and 12.58 times for the standalone PMS system (0.0063 min^−1^), the standalone CuFe_2_O_4_ system (0.0038 min^−1^) and the CuFe_2_O_4_/PDS system (0.0090 min^−1^), respectively. Therefore, the subsequent experiments adopted the CuFe_2_O_4_/PMS system to further investigate the degradation process of OFL.

#### 3.2.2. Effects of Reaction Parameters on the Degradation of OFL

The activation of PMS and the degradation performance of the catalyst depend, not only on their physical and chemical properties, but also on other reaction conditions and parameters. Therefore, the influence of factors, including catalyst dosage, PMS concentration and initial pH, on the removal efficiency of OFL was studied systematically.

**Figure 3:**
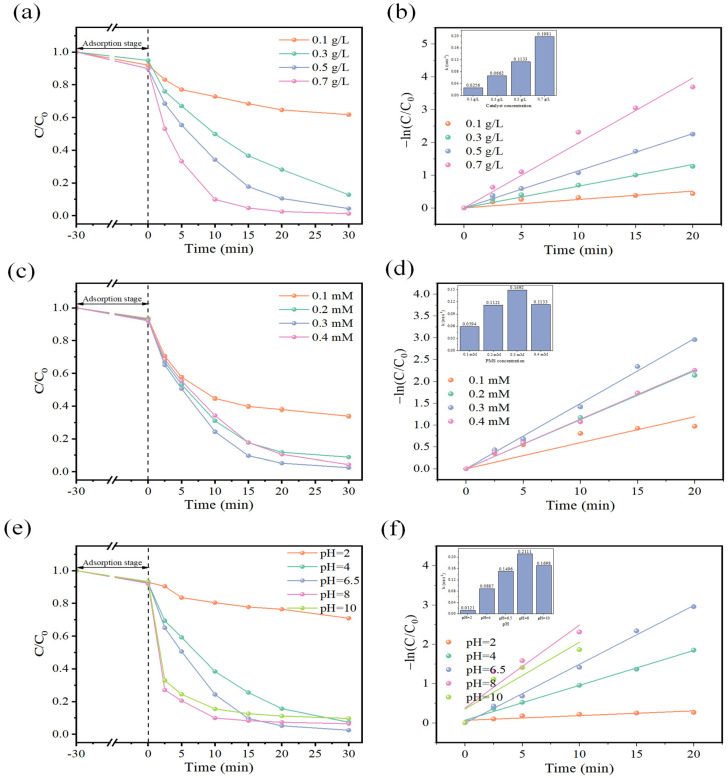
Effects of (**a**,**b**) catalyst dosage, (**c**,**d**) PMS concentration, (**e**,**f**) initial pH on OFL degradation and its corresponding kinetics in the CuFe_2_O_4_/PMS system ([OFL]_0_ = 10 mg/L, [CuFe_2_O_4_]_0_ = 0.5 g/L except panel (**a**), [PMS]_0_ = 0.4 mM except panel (**c**), pH unadjusted except panel (**e**)).

The degradation efficiency of OFL under different catalyst dosages and its corresponding kinetics are shown in Figure 3a,b. When the dosage of CuFe_2_O_4_ is 0.1 g/L, the removal efficiency of OFL is only 38.27%. However, when the catalyst dosage is increased to 0.7 g/L, OFL is almost completely degraded with a degradation efficiency of 98.76%, followed by the highest rate constant of 0.1981 min^−1^. This is attributed to the fact that the increased catalyst dosage provides more active sites, which enable the activation of PMS by generation of more free radicals [25].

Figure 3c,d illustrates the effect of different PMS concentrations on the removal efficiency of OFL and its corresponding first-order kinetics. At PMS concentrations ranging from 0.1 to 0.3 mM, the removal efficiency of OFL increased steadily from 61.18% to 97.53% with increasing PMS concentration, followed by the increase in rate constants from 0.0594 min^−1^ to 0.1492 min^−1^. However, only 6.00% of the degradation rate is enhanced when the PMS dosage rises from 0.2 to 0.3 mM. The slight increase can be attributed to the limited number of active sites provided by the catalyst, which may not be sufficient to activate abundant PMS molecules, resulting in a slow rate of reactive species generation. However, after further increasing the PMS concentration to 0.4 mM, there is a slight decrease in the degradation rate of OFL, with a relatively low rate constant of 0.1133 min^−1^. This can be attributed to the fact that high concentrations of PMS can lead to self-deactivation of radicals or competitive reactions, resulting in the formation of less active radicals [26], which in turn affects the catalytic performance.

In addition to catalyst dosage and PMS concentration, the initial pH is also a critical influencing factor in the system. Figure 3e illustrates the degradation efficiency of OFL in the CuFe_2_O_4_/PMS system with different initial pH values. Under strong acidic conditions, the degradation of OFL is significantly inhibited, with a removal rate of only 29.09% at pH 2.0. This inhibition is mainly attributed to the destruction of active sites and surface functional groups of the catalyst under extremely acidic conditions [27]. Meanwhile, the pK_a_ value of PMS is 9.4 (Equation (2)), at which point it is mainly in the form of HSO_5_^−^. Acidic conditions favor the formation of a strong hydrogen bond between the H^+^ in the solution and the O–O group of HSO_5_^−^, stabilizing the HSO_5_^−^ species and inhibiting its interaction with other positively charged oxidizing species on the surface. The isoelectric potential of CuFe_2_O_4_ is speculated to be around 8 [28], indicating that the surface of CuFe_2_O_4_ is positively charged when pH < 8. When increasing the initial pH continuously, the force between HSO_5_^−^ and H^+^ in solution weakened and the electrostatic attraction between PMS and CuFe_2_O_4_ was enhanced [29], facilitating the activation of PMS by CuFe_2_O_4_ with an improvement of catalytic performance (as well as the corresponding kinetics) until it reached the optimum level at pH = 6.5. It is worth noting that as we further increase the initial pH value, although the final removal rate of OFL gradually decreases, Figure 3f reveals that there is a rapid reaction within the first 10 min after the addition of PMS under alkaline conditions. The pseudo-first order kinetics constants for initial pH (8 and 10) are 0.2111 min^−1^ and 0.1698 min^−1^, respectively, both higher than the value of 0.1496 min^−1^ for the neutral initial solution. This can be attributed to the reaction between SO_4_^•−^ and water or OH^−^ in the alkaline condition, leading to the generation of •OH (Equations (3) and (4)). Compared to SO_4_^•−^, •OH has a shorter lifespan [30]. Therefore, some degradation effects can be observed within the first 10 min of the reaction. In alkaline conditions, HSO_5_^−^ exists mainly as SO_2_^5−^, according to Equation (2). When the pH is higher than the isoelectric potential of CuFe_2_O_4_ (around 8), the negative charge on the catalyst is not conducive to the binding of SO_2_^5−^ and HSO_5_^−^, resulting in a slight decrease in degradation efficiency. When monitoring the pH changes in different systems (Figure S3), it can be observed that, except in highly alkaline and acidic environments, the addition of PMS leads to transient changes and the solution is stable near neutral pH, indicating a certain buffering capacity of the system.

(2) $${\mathrm{HSO}}_{5}^{-}↔H^{+}{+\mathrm{SO}}_{5}^{2-},\;\mathrm{pKa}=9.4$$

(3) $${\mathrm{SO}}_{4}^{•-}+{\mathrm{OH}}^{-}\to •{\mathrm{OH}+\mathrm{SO}}_{4}^{2-}$$

(4) $${\mathrm{SO}}_{4}^{•-}+H_{2}O\to •\mathrm{OH}+{\mathrm{SO}}_{4}^{2-}+H^{+}$$

#### 3.2.3. Optimization of OFL Removal

To better understand the effect of catalyst dosage, PMS concentration and initial pH in the CuFe_2_O_4_/PMS system, the orthogonal experiment was designed by the Box-Behnken design (BBD) with response surface methodology (RSM) (Design Expert 13.0.1.0 software). Three process parameters were selected at three different levels based on single-factor experiments. 

Through 17 random experiments generated by BBD, the interactive effects of three variables, namely catalyst dosage (A), PMS concentration (B) and pH (C), on the removal efficiency of OFL were investigated. From Table S1, it can be observed that the CuFe_2_O_4_/PMS system achieved OFL removal rates ranging from 8.65% to 99.36% within the selected parameter range, and the predicted values closely matched the actual values. Regression analysis was performed to fit these responses without the need for transformation, resulting in the final model as follows:

$$\begin{array}{l} Y=-79.11+173.78A+215.97B+24.07C+150.7\mathrm{AB}\\ +1.64\mathrm{AC}+14.83\mathrm{BC}-183.36A^{2}-618.87B^{2}-1.99C^{2} \end{array}$$

where the Y value represents the predicted OFL degradation efficiency, while A, B and C represent the values of the three influencing factors. The positive and negative signs indicate the respective positive or negative influence on the OFL degradation efficiency.

The specific significance of the model was confirmed by the F-value (59.66) and the *p*-value (<0.0001) from analysis of variance (ANOVA), indicating its significance. Furthermore, the *p*-value of lack-of-fit was 0.0555, which is not significant, further validating the significance of the model. The R-squared value and adjusted R-squared value of the model were 0.9871 and 0.9706, respectively, suggesting a good fit between the model predictions and experimental values. The standard deviation (Std. Dev.) was 5.21, indicating the excellent accuracy and reliability of the model. Additionally, the Adeq precision was 23.549, significantly greater than 4, indicating a sufficient signal.

**Figure 4:**
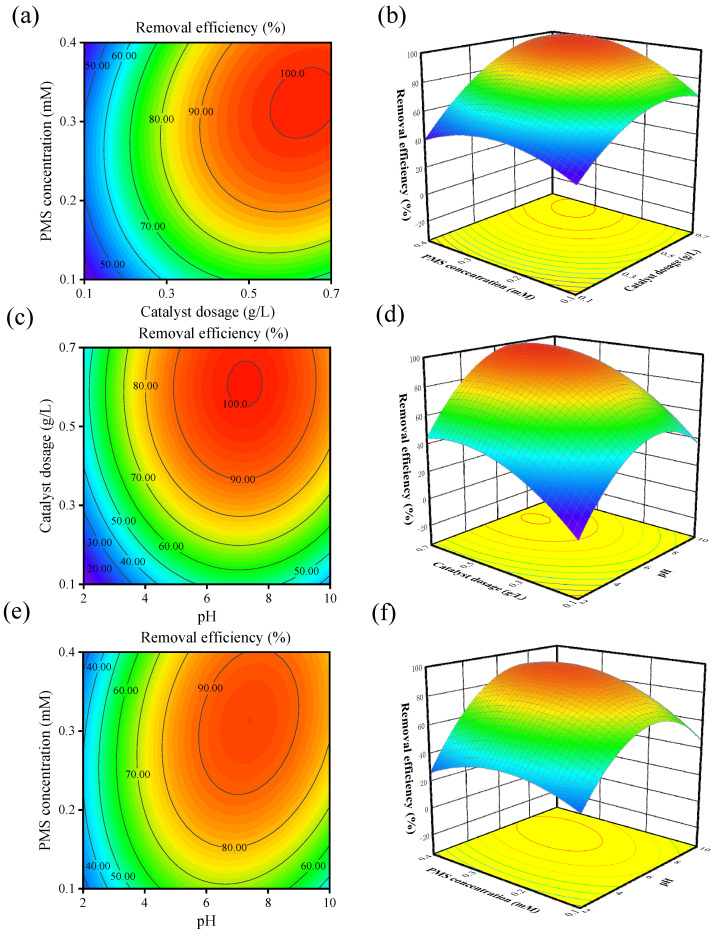
The contour and surface of OFL removal efficiency about PMS concentration and catalyst dosage (**a**,**b**), catalyst dosage and pH (**c**,**d**), PMS concentration and pH (**e**,**f**).

2D contour plots and 3D surface plots were used to investigate the interactive effects of catalyst dosage, PMS concentration and pH, as well as the individual factors, on the OFL removal rate. The elliptical shape of the contour plots in Figure 4a,c,e indicates a significant interaction among different influencing factors. Figure 4a,b displays contour and response surface plots for catalyst dosage and PMS concentration. The lowest degradation rate (36.03%) was observed when the catalyst dosage was 0.1 g/L and the PMS concentration was 0.1 mM. As the catalyst dosage increased from 0.1 g/L to 0.7 g/L and the PMS concentration increased from 0.1 mM to 0.4 mM, the OFL removal rate gradually increased. The increase in catalyst dosage improved the accessibility of the active sites, while the increase in PMS concentration enhanced the activation performance and the rate of free radical generation. When the catalyst dosage was 0.56 g/L, the OFL removal rate reached 100% at a PMS concentration of 0.29 mM. However, after reaching the optimum OFL removal rate, the degradation rate decreased with increasing catalyst dosage and PMS concentration due to the unfavorable consumption of active free radicals. Figure 4c,d shows that with a PMS concentration of 0.25 mM, the optimum degradation efficiency of OFL was achieved at pH = 6.9 with increasing catalyst concentration. Figure 4e,f, analyzed the influence of pH and PMS concentration on the OFL removal rate with a fixed catalyst dosage of 0.4 g/L. It was found that the removal rate of OFL is hard to reach 100% when the catalyst dosage is low, indicating an insufficient supply of active sites. Figure 4c–e demonstrate a strong degradation performance near neutral pH. Analysis of the F-values of the different factors in ANOVA identified catalyst dosage and pH as the main factors affecting OFL degradation, while PMS concentration had a lesser effect.

To verify the practical application of RSM-BBD in the CuFe_2_O_4_/PMS system for OFL removal efficiency, the optimal conditions predicted by the model were as follows: catalyst dosage = 0.66 g/L, PMS concentration = 0.38 mM and pH = 6.53. Under these conditions, the degradation efficiency reached 100%, and the model’s desirability value was 1.0. To validate the accuracy of the model predictions, three parallel experiments were conducted based on these conditions, resulting in removal rates of 99.49%, 100% and 98.87%, respectively. This confirms that the model can accurately predict the optimal operating conditions for the experiments.

#### 3.2.4. Effect of Water Matrices on OFL Removal

**Figure 5:**
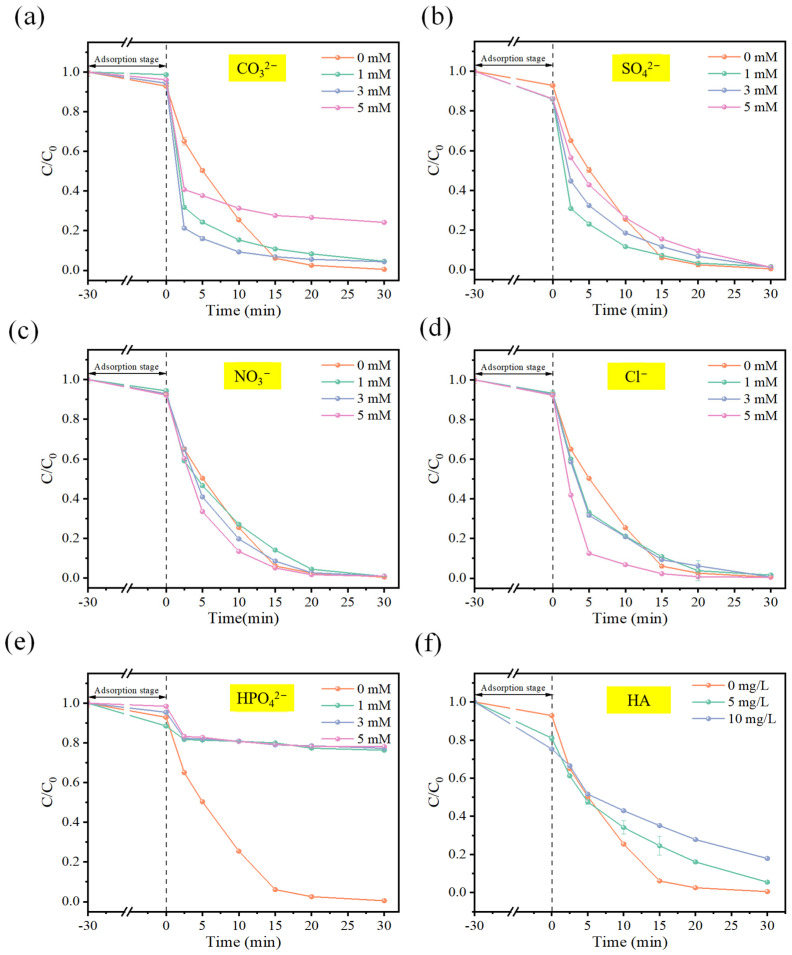
Effects of (**a**) CO_3_^2−^, (**b**) SO_4_^2−^, (**c**) NO_3_^−^, (**d**) Cl^−^, (**e**) HPO_4_^2−^ and (**f**) HA on the degradation of OFL. (pH unadjusted, [OFL]_0_ = 10 mg/L, [CuFe_2_O_4_]_0_ = 0.66 g/L, [PMS]_0_ = 0.38 mM).

The effect of common anions (CO_3_^2−^, SO_4_^2−^, NO_3_^−^, Cl^−^ and HPO_4_^2−^) at concentrations of 1, 3 and 5 mM on the efficiency of OFL degradation in the CuFe_2_O_4_/PMS process was investigated. The results from Figure 5 indicate that the presence of SO_4_^2−^, NO_3_^−^ and Cl^−^ has only a minor effect on OFL degradation. Figure 5a demonstrates that the presence of CO_3_^2−^ led to the rapid activation of PMS in the system within the first 2.5 min. This is partly due to the hydrolysis of CO_3_^2−^ upon introduction into the water, which generates HCO_3_^−^ and OH^−^ (Equation (5)). The production of OH^−^ activates PMS under alkaline conditions, leading to a rapid reaction. Additionally, low concentrations of carbonate ions can act as accelerators for PMS decomposition and simultaneously generate radicals [31], thereby promoting oxidative reactions. However, when the concentration of CO_3_^2−^ reaches 5 mM, an excessive formation of CO_3_^•−^ occurs (Equations (6) and (7)), resulting in a decrease in the concentration of radicals and, consequently, a decrease in degradation efficiency. Figure S4 reveals that the first-order kinetics of Cl^−^ at a concentration of 5 mM are significantly higher than those observed in the sole CuFe_2_O_4_/PMS system. This promoting effect can be attributed to the preferential reaction of Cl• with certain pollutants, particularly electron-rich compounds, such as OFL, leading to the formation of adsorbed organohalides by combining with unsaturated bonds in OFL. This, in turn, facilitates further degradation of OFL [32]. Furthermore, it can be observed that HPO_4_^2−^ has a pronounced inhibitory effect (Figure 5e). This is primarily because HPO_4_^2−^ acts as a potential ligand for transition metals, especially on the goethite surface, displaying strong binding capabilities. As a result, it not only competes with OFL for active adsorption sites on the surface, but also forms an inner-sphere complex by displacing hydroxyl groups on the catalyst surface with metal ions [26].

Humic acids (HA), as one of the representatives of natural organic matter (NOM), were studied to evaluate the effect of NOM on OFL removal in the CuFe_2_O_4_/PMS system. As depicted in Figure 5f, the addition of HA significantly inhibited the degradation of OFL. The degradation rate gradually decreased with increasing concentration of HA. When 5 mg/L and 10 mg/L of HA were introduced into the system, the removal efficiency of OFL decreased from 99.49% in the control experiment to 94.56% and 82.14%, respectively. This is primarily due to the ability of HA to act as a free radical scavenger. It competes with OFL to react with active radicals. Additionally, the phenolic hydroxyl and carboxyl groups of HA may also adsorb onto the CuFe_2_O_4_ surface, occupying active sites and inhibiting the progress of the reaction [33].

(5) $${\mathrm{CO}}_{3}^{2-}+H_{2}O\to {\mathrm{HCO}}_{3}^{-}+{\mathrm{OH}}^{-}$$

(6) $${\mathrm{HCO}}_{3}^{-}+{\mathrm{SO}}_{4}^{•-}\to {\mathrm{CO}}_{3}^{•-}+{\mathrm{HSO}}_{4}^{-}$$

(7) $${\mathrm{HCO}}_{3}^{-}+•\mathrm{OH}\to {\mathrm{CO}}_{3}^{•-}+H_{2}O$$

#### 3.2.5. Practical Application in CuFe_2_O_4_/PMS System

**Figure 6:**
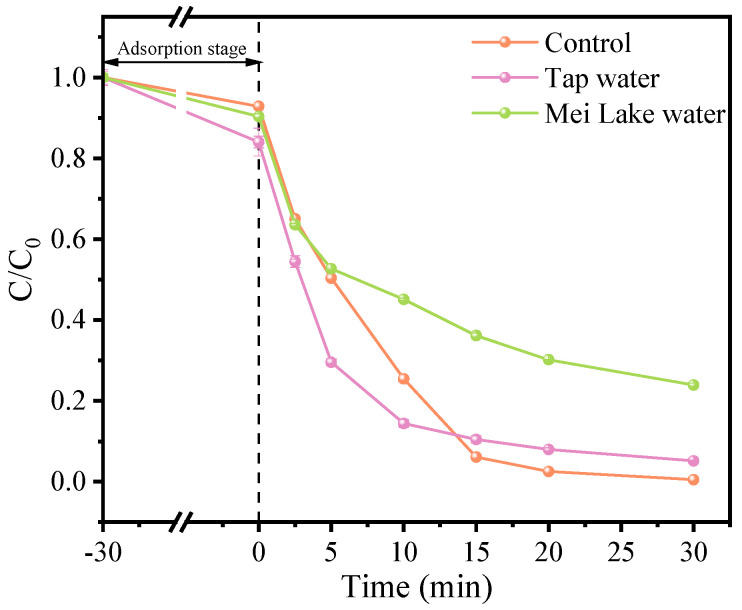
Effects of tap water and Mei Lake water on OFL degradation in CuFe_2_O_4_/PMS process. (pH unadjusted, [OFL]_0_ = 10 mg/L, [CuFe_2_O_4_]_0_ = 0.66 g/L, [PMS]_0_ = 0.38 mM).

As shown in Figure 6, the degradation capacity of the CuFe_2_O_4_/PMS system for OFL in actual water bodies was investigated with tap water and Mei Lake water (taken from Zhengzhou University). Moreover, the pH of Mei Lake water is about 7.8. The total carbon (TC), inorganic carbon (IC) and total organic carbon (TOC) of Mei Lake water were 46.21 mg/L, 27.21 mg/L and 19.00 mg/L, respectively.

In tap water, the presence of chlorine leads to the generation of Cl•, which probably contributed to the promotion of OFL degradation in the first 10 min, which is consistent with the result of Figure 5d. After 30 min, the degradation efficiency of OFL reaches 94.85% in tap water, which has a slightly inhibitory effect compared to the control system. Using Mei Lake water as a background sample, the degradation efficiency of OFL is 76.07%. It is speculated that Mei Lake water contains a certain amount of HCO_3_^−^ (based on the IC of 27.21 mg/L) and some naturally dissolved organic matter (based on the TOC of 19.00 mg/L), which reduced the concentration of free radicals to some extent, resulting in a decrease in the degradation efficiency of OFL. Herein, the CuFe_2_O_4_/PMS system performs well in OFL degradation in actual water bodies even though it contains a certain concentration of inorganic anions and NOM.

### 3.3. Stability of CuFe_2_O_4_

**Figure 7:**
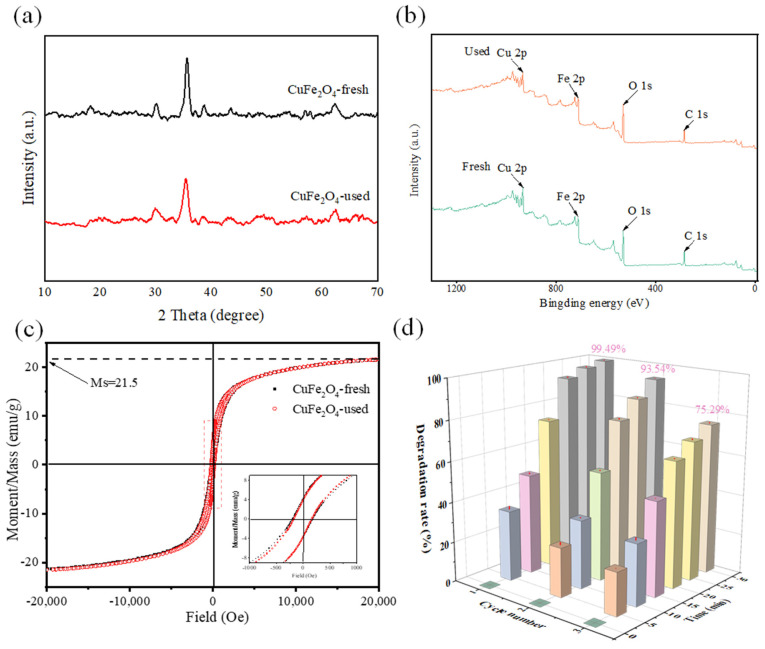
(**a**) XRD patterns, (**b**) XPS for the overall survey, and (**c**) VSM for the overall survey of the fresh and used CuFe_2_O_4_ nanoparticles in the CuFe_2_O_4_/PMS system. (**d**) Recycling experiments of CuFe_2_O_4_/PMS system OFL degradation.

The stability of catalysts is a crucial indicator in practical applications. Recycling experiments were therefore carried out to evaluate the stability of the prepared catalyst. Then, the fresh and used samples were detected with the help of XRD, VSM and XPS, as shown in Figure 7. Clearly, the specific diffraction peaks of used CuFe_2_O_4_ have no significant changes compared to that of the fresh CuFe_2_O_4_ sample (Figure 7a), indicating that CuFe_2_O_4_ possesses good crystallinity and structural stability. In addition, the similar XPS survey spectra of fresh and used CuFe_2_O_4_ samples (Figure 7b) indicate the stability of the bulk structure of CuFe_2_O_4_ consisting of Cu, Fe and O elements.

Furthermore, as a magnetic catalyst, the magnetic strength after use is crucial for its recovery and reuse. The magnetic properties of CuFe_2_O_4_ before and after use were evaluated using VSM. As observed in Figure 7c, the magnetic hysteresis loops of the used CuFe_2_O_4_ showed no apparent changes compared to that of the fresh one, and both exhibited a saturation magnetization intensity of 21.5 emu/g, indicating that the CuFe_2_O_4_ catalyst retained excellent saturation magnetism after recovery and reuse.

Additionally, the recycling experiments of the CuFe_2_O_4_/PMS system with the magnetic separation recovery of CuFe_2_O_4_ were shown in Figure 7d. After three recycles, the removal efficiency of OFL decreased from 99.49% to 75.29%, indicating that CuFe_2_O_4_/PMS still exhibited strong catalytic activity after three time cycles. The slight decrease in efficiency may be attributed to the adsorption of degradation intermediates on the active sites of the catalyst, the leaching of metal ions and catalyst aggregation [34]. 

### 3.4. Mechanisms of PMS Activation by CuFe_2_O_4_

**Figure 8:**
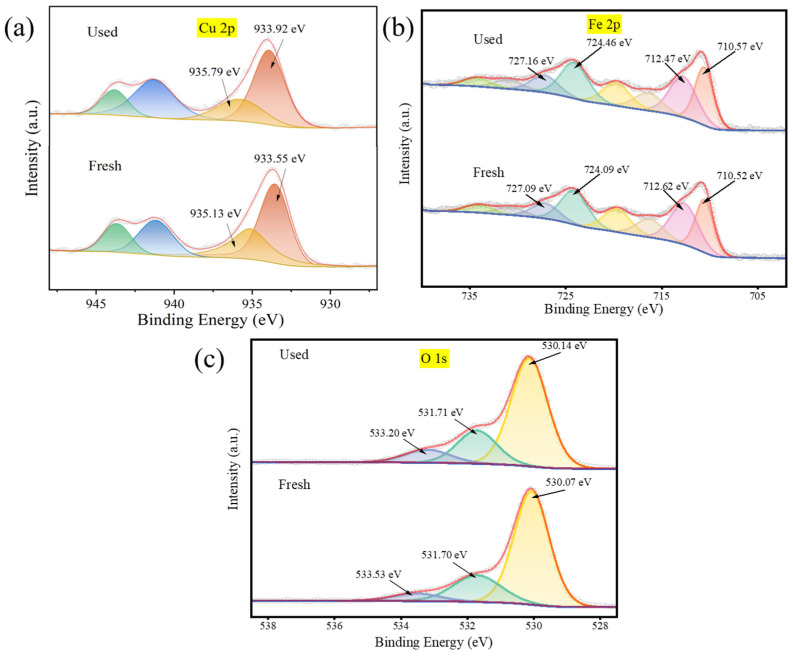
XPS spectra of (**a**) Cu 2p, (**b**) Fe 2p and (**c**) O 1s for fresh and used CuFe_2_O_4_ catalysts.

To explore the possible catalytic degradation mechanisms of the CuFe_2_O_4_/PMS system, XPS was examined to analyze the chemical states of elements in fresh and used CuFe_2_O_4_ samples, as shown in Figure 8, with all binding energies referenced to C 1s at 284.8 eV. Figure 8a shows the Cu 2p XPS spectra, the characteristic peaks at 933.55 eV in a fresh one corresponded to the Cu(I), accounting for 64.05% of the total peaks. Furthermore, the peaks at 935.13 eV corresponded to the Cu(II), representing 35.95% of the total peaks. In the XPS spectrum of the used Cu 2p, the proportion of Cu(I) increased to 70.16% and Cu(II) decreased to 29.84%, indicating that the Cu species are involved in the degradation reaction. Moreover, the slight blueshift of the Cu(II) peak suggests an increase in the Cu(II) oxidation state after the reaction with PMS [35]. The slight blueshift of the Cu(I) indicates the generation of partial oxygen vacancies after the reaction [36], resulting in oxygen loss and the generation of superoxide radicals, consistent with the ESR characterization results.

Figure 8b presents the Fe 2p XPS spectrum, where the peaks observed at 710.2 eV and 724.09 eV are attributed to Fe(II), while the peaks at 712.62 eV and 727.09 eV correspond to Fe(III). The oxidation state of Fe remained unchanged before and after the reaction, possibly due to the weaker catalytic activity of Fe towards PMS in a neutral environment [37], consistent with the earlier results from the Fe_2_O_3_ degradation experiment.

The XPS spectra of the O 1s are displayed in Figure 8c. The characteristic peaks around 530.14 eV, 531.71 eV and 533.20 eV derive from lattice oxygen, physically or chemically adsorbed oxygen in surface water and hydroxyl oxygen on the metal surface. After the reaction, these peaks account for 67.72%, 22.16% and 10.11%, respectively, compared to the pre-reaction values of 70.91%, 22.64% and 6.45%. The decrease in lattice oxygen corresponds to an increase in surface hydroxyl oxygen, indicating strong hydroxylation on the CuFe_2_O_4_ catalyst surface during the reaction [38]. Furthermore, the abundant surface hydroxyl groups enhance the dispersion of the catalyst, thereby increasing the effective reaction area between the catalyst and the pollutants [39] and further strengthening the catalytic degradation of OFL.

**Figure 9:**
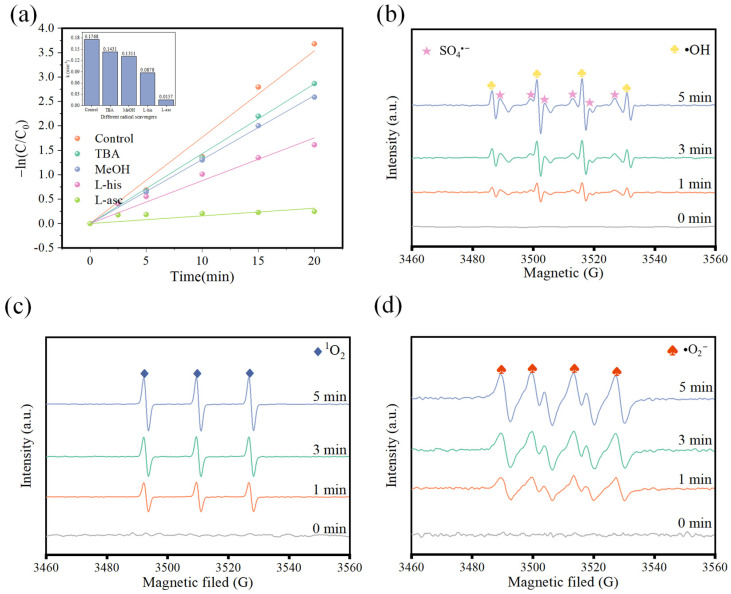
(**a**) First-order kinetics constants for different radical scavengers; ESR spectrum at reaction times of 0, 1, 3, 5 min in the CuFe_2_O_4_/PMS system: (**b**) SO_4_^•−^ and •OH, (**c**) ^1^O_2_ and (**d**) •O_2_^−^. Reaction conditions: (pH unadjusted, [OFL]_0_ = 10 mg/L, [CuFe_2_O_4_]_0_ = 0.66 g/L, [PMS]_0_ = 0.38 mM).

In order to further investigate the types of reactive oxygen species (ROS) generated by the CuFe_2_O_4_/PMS system, a comprehensive quenching experiment and ESR measurements were conducted as shown in Figure 9. Generally, SO_4_^•−^ and •OH are commonly regarded as key species in the activation process of PMS. MeOH was used as a SO_4_^•−^ and •OH scavenger because of the similar rate constants of MeOH and the radical, while TBA was commonly employed as the •OH scavenger with a higher rate constant (k = (3.8–7.6) × 10^8^ mol L^−1^ s^−1^) of •OH than that of MeOH ((1.6–7.7) ×10^7^ mol L^−1^ s^−1^) [40]. Thus, the contributions of SO_4_^•−^ and •OH during the degradation process could be effectively identified. As seen in Figure S5, the degradation efficiency of OFL has little difference in the presence of MeOH and TBA compared to the control system. However, there is a distinct decrease of rate constants in both systems compared to the control system (Figure 9a). In detail, the system with MeOH shows a stronger inhibitory effect (0.1311 min^−1^) compared to the system with TBA (0.1430 min^−1^). Simultaneously, signals corresponding to hydroxyl radicals and sulfate radicals were detected during the ESR measurement in Figure 9b. Moreover, it was evident that the signal values gradually increased from 0 to 5 min as the reaction progressed, in agreement with the results obtained from the quenching experiments. These results indicate that both SO_4_^•−^ and •OH are generated and play a role in the degradation process to some extent. In addition, L-histidine (L-his) and L-ascorbic acid (L-asc) are considered to be the scavengers of singlet oxygen (^1^O_2_) and superoxide radicals (•O_2_^−^), respectively. In the presence of L-his, the removal efficiency of OFL decreased by 12.82% with a relative low rate constant of 0.0878 min^−1^ compared to the control system; also, the intensity of the ^1^O_2_ signals in CuFe_2_O_4_/PMS was much higher over time (Figure 9c), indicating the generation of ^1^O_2_ during the process. As shown in Figure 9d, the signal of superoxide radicals was largely detected in the ESR test. In addition, with the introduction of L-asc into the system, the degradation rate of OFL decreased from 99.49% to 24% and the corresponding rate constant decreased to 0.016 min^−1^, indicating that •O_2_^−^ was generated and played a key role in the degradation process. All in all, SO_4_^•−^, •OH, •O_2_^−^ and ^1^O_2_ are involved in the degradation of OFL in the system, with •O_2_^−^ acting as the primary active species in the CuFe_2_O_4_/PMS system.

Based on a series of experiments and characterization results, the potential mechanisms of CuFe_2_O_4_ activation of PMS were proposed. In this process, the cyclic regeneration of ≡Cu(I)/≡Cu(II) and ≡Fe(III)/≡Fe(II) played a key role in the generation of active radicals. Initially, upon PMS activation, Cu(I) and Fe(II) can be oxidized to Cu(II) and Fe(III) by HSO_5_^−^ with the generation of SO_4_^•−^. In addition, the •OH can be generated by the reaction between SO_4_^•−^ and H_2_O or OH^−^ in solution according to Equations (2) and (3). The possible sources of •O_2_^−^ are as follows: (1) PMS decomposition generates SO_5_^2−^, which, when activated by the catalyst, reacts with water to produce •O_2_^−^ and H+ (Equations (12) and (13)); (2) Cu(I) and Fe(II) in CuFe_2_O_4_ transfer electrons to O_2_ and utilize the abundant oxygen vacancies within the CuFe_2_O_4_ lattice to reduce O_2_ to •O_2_^−^; (3) furthermore, persistent semiquinone-type free radicals generated by PMS activation can transfer electrons to O_2_, leading to the production of •O_2_^−^ [41]. The oxidation–reduction potentials of O_2_/•O_2_^−^, Cu(II)/Cu(I) and Fe(III)/Fe(II) are −0.33, 0.17 and 0.77 V vs. RHE, respectively [42]. Therefore, thermodynamically, the presence of •O_2_^−^ facilitates the reduction of Cu(II) and Fe(III) to Cu(I) and Fe(II), achieving continuous cyclic regeneration in the system (Equations (8)–(11)). Additionally, the system also produces singlet oxygen (^1^O_2_), a non-free radical species, which contributes to OFL degradation. The abundant presence of •O_2_^−^ promotes the generation of ^1^O_2_, while SO_5_^•−^ and HSO_5_^−^ can also produce ^1^O_2_ under the effect of the catalyst (Equations (12)–(17)).

(8) $$\equiv {\mathrm{Cu}(I)+\mathrm{HSO}}_{5}^{-}\to \equiv \mathrm{Cu}(\mathrm{II})+{\mathrm{SO}}_{4}^{•-}{+\mathrm{OH}}^{-}$$

(9) $$\equiv {\mathrm{Fe}(\mathrm{II})+\mathrm{HSO}}_{5}^{-}\to \equiv \mathrm{Fe}(\mathrm{III})+{\mathrm{SO}}_{4}^{•-}{+\mathrm{OH}}^{-}$$

(10) $$\equiv \mathrm{Cu}(\mathrm{II})+•O_{2}^{-}\to \equiv \mathrm{Cu}(I)+O_{2}$$

(11) $$\equiv \mathrm{Fe}(\mathrm{III})+•O_{2}^{-}\to \equiv \mathrm{Fe}(\mathrm{II})+O_{2}$$

(12) $${\mathrm{SO}}_{5}^{2-}+H_{2}O\to •O_{2}^{-}{+\mathrm{SO}}_{4}^{2-}+H^{+}$$

(13) •O2−+2H2O→H2O2+2OH−+O21

(14) •O2−+•OH→OH−+O21

(15) •O2−+•O2−+2H+→H2O2+O21

(16) 2SO5•−+H2O→1.5O21+2HSO4−

(17) HSO5−+SO52−→O21+SO42−+HSO4−

### 3.5. Intermediates of OFL Degradation and Toxicity Assessment

**Figure 10:**
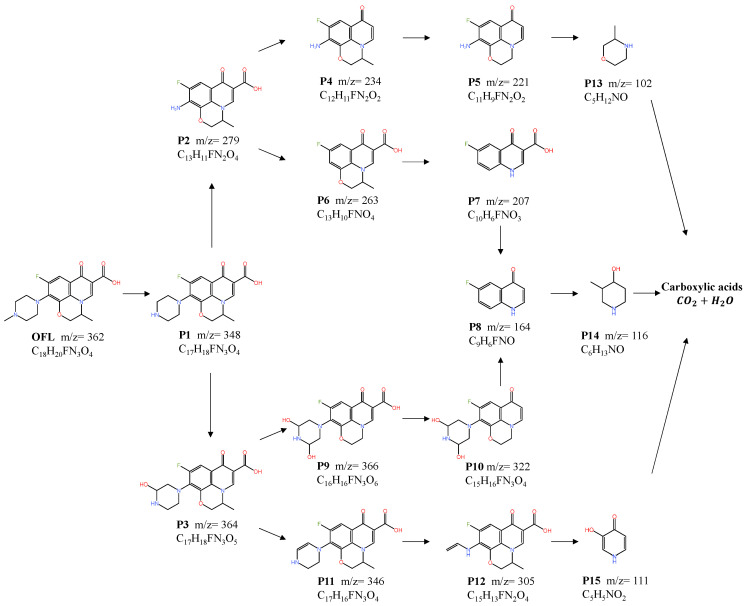
The possible degradation pathways of OFL in the CuFe_2_O_4_/PMS system.

In order to elucidate the degradation pathway of OFL, UPLC-QTOF-MS/MS was employed to determine the intermediates generated during the degradation of OFL in the CuFe_2_O_4_/PMS system. Based on the ESI-QTOF-MS/MS results and the prior literature, the possible degradation intermediates were detected and the molecular structures are listed in Table S2. Figure 10 depicts the potential degradation pathways of OFL in the CuFe_2_O_4_/PMS system. Initially, the methyl group of the piperazinyl substituent is cleaved under the attack of active radials to form P1 (*m*/*z* = 348). Through the synergistic effect of free radicals and non-free radicals, the C-N bond on the piperazine ring cleaves, leading to the formation of P2 (*m*/*z* = 279). Subsequently, the C-C bond connecting the carboxyl group becomes the target, resulting in the removal of the carboxyl group from P2 and subsequent dehydrogenation to form P4 (*m*/*z* = 234). In addition, the methyl group on the oxazolidinone ring is further eliminated to produce P5 (*m*/*z* = 221). Simultaneously, under the synergistic action of radicals and non-radicals, continuous attacks on nitrogen-containing heterocycles and benzene rings lead to the cleavage of C-C and C-N bonds, resulting in the formation of P13 (*m*/*z* = 102) [43]. Furthermore, P2 can undergo further deamination and, under the attack from •O_2_^−^, the quinoxaline ring will be cleaved to form P7 (*m*/*z* = 207). Subsequently, the hydroxylation and decarboxylation of P15 (*m*/*z* = 164) give rise to P8 (*m*/*z* = 164), which then replaces the carbon–oxygen double bond to form P14 (*m*/*z* = 116).

Additionally, the pyrimidine ring on P1 can undergo hydroxylation, generating P3 (*m*/*z* = 364). It can then follow two different pathways. The first pathway involves further hydroxylation to form P9 (*m*/*z* = 366) and subsequent decarboxylation and dehydrogenation to form P10 (*m*/*z* = 322). This degradation process is similar to previous reports [44]. Then, through carboxylation, it transforms into P8 and P14, becoming small molecular intermediates. The second pathway involves the dehydroxylation of P3, resulting in the opening of the nitrogen-containing heterocycle under continuous attacks from active species, leading to the formation of P12 (*m*/*z* = 305). Further degradation results in the complete cleavage and elimination of the pyrimidine and quinoxaline rings, together with decarboxylation, resulting in the formation of P15 (*m*/*z* = 111). 

Ultimately, through the combined action of various reactive species, the intermediates are continuously attacked, mineralized and decomposed into organic small molecules, CO_2_ and H_2_O.

### 3.6. Toxicity Evaluation of Products in Degradation Pathways

**Figure 11:**
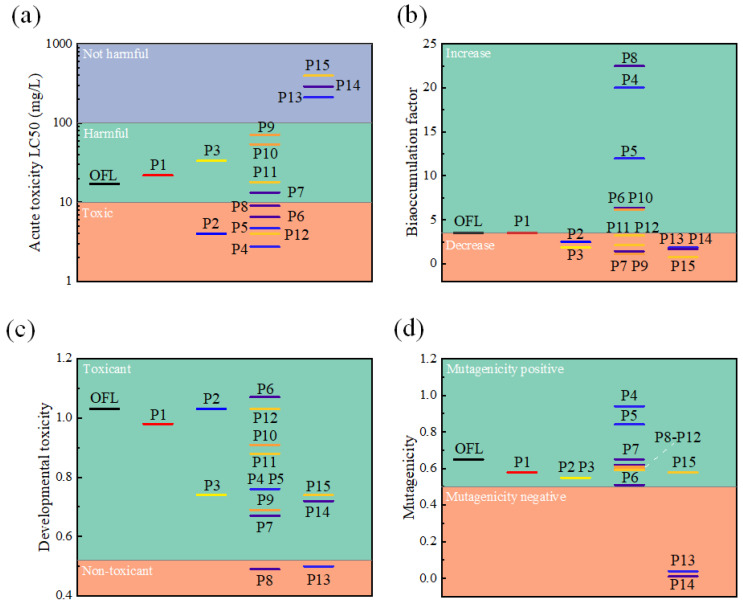
The QSAR predictions of OFL and intermediate products: (**a**) acute toxicity (Daphnia magna LC_50_-48 h), (**b**) bioaccumulation factor, (**c**) developmental toxicity and (**d**) mutagenicity.

The toxicity assessment software T.E.S.T. 5.1.2.0 was utilized to analyze the acute toxicity, mutagenicity, developmental toxicity and bioaccumulation factor of OFL and its intermediate products (Ps). Acute toxicity was predicted by the median lethal concentration (LC_50_) of Daphnia magna after 48 h, as depicted in Figure 11a. Although some intermediate products exhibit higher toxicity than OFL during degradation, the intermediate products P13–15 are directly transformed into harmless substances, indicating a decrease in acute toxicity as the degradation progresses. The bioaccumulation factor of OFL is 3.53 (Figure 11b), and the bioaccumulation factors of P4-P6, P8 and P10 remain higher than OFL, suggesting that these substances are more prone to accumulate in organisms compared to OFL. However, most of the intermediate products have lower bioaccumulation factors than OFL after further degradation, indicating that thorough degradation helps to reduce their bioaccumulation potential. Furthermore, from Figure 11c, it can be observed that the developmental toxicity of intermediate products, except for P6, is lower than that of OFL, with P8 and P13 exhibiting non-toxic results. Moreover, the simulation results for mutagenicity and developmental toxicity are similar; except for P4 and P5, the mutagenicity of other intermediate products is lower than that of OFL, and P13 and P14 become non-mutagenic products. As a result, the present CuFe_2_O_4_/PMS system can efficiently remove OFL and reduce the negative impact of OFL on the environment.

## 4. Conclusions

In this study, magnetic CuFe_2_O_4_ was successfully obtained by an improved sol–gel method for PMS activation and OFL degradation. Approximately 100% of OFL could be removed under optimal reaction conditions that were evaluated by response surface methodology with the Box-Behnken design: a CuFe_2_O_4_ dosage of 0.66 g/L, a PMS concentration of 0.38 mM and a pH of 6.53 without adjustment in solution. Except HPO_4_^2−^, the other inorganic anions, humic acid and actual water bodies (Mei Lake and tap water) have little effect on the performance of the CuFe_2_O_4_/PMS system, indicating the outstanding anti-interference ability of the system. The outstanding degradation activity contributed to the continuous generation of SO_4_^•−^, •OH, •O_2_^−^ and ^1^O_2_ under cyclic transformation between Cu(II)/Cu(I) and Fe(III)/Fe(II) on the surface-bound CuFe_2_O_4_. About 15 types of intermediates were identified and the possible degradation pathway was predicted. At the same time, the degradation process is considered an environmentally friendly process according to the less toxic assessment of the final intermediates. The study of the fluoroquinolones degradation pathway in the CuFe_2_O_4_/PMS system provides a reference for PMS-based advanced oxidation processes. 

## Data Availability

The data presented in this study are available on request from the corresponding author.

## Supplementary Materials

The following supporting information can be downloaded at https://www.mdpi.com/article/10.3390/toxics12100731/s1, Figure S1: the degradation rate of OFL for various samples ([pH]_0_ = 6.5 (unadjusted), [OFL]_0_ = 10 mg/L, [catalyst]_0_ = 0.5 g/L, [PMS]_0_ = 0.4 mM); Figure S2: the EIS curves for CuFe_2_O_4_, Fe_2_O_3_, and CuO; Figure S3: the pH change tendencies for the different initial pH values; Figure S4: the first-order kinetics constants for different inorganic anions; Figure S5: the effect of radical scavengers on OFL degradation efficiency in the CuFe_2_O_4_/PMS system. Reaction conditions: (pH unadjusted, [OFL]_0_ = 10 mg/L, [CuFe_2_O_4_]_0_ = 0.66 g/L, [PMS]_0_ = 0.38 mM). Table S1: the experimental design matrix generated by BBD; Table S2: the proposed intermediates of OFL degradation over CuFe_2_O_4_/PMS system. Text S1: materials and reagents; Text S2: the characterizations of CuFe_2_O_4_; Text S3: the evaluation of the catalytic performance.
