# Supplementary material for: Efficient Degradation of Ofloxacin by Magnetic CuFe2O4 Coupled PMS System: Optimization, Degradation Pathways and Toxicity Evaluation
Source: Toxics. 2024 Oct 10;12(10):731. doi: 10.3390/toxics12100731 (PMC11510951; doi:10.3390/toxics12100731)
Supplement: Supplementary file 1 [file toxics-12-00731-s001.zip › toxics-3199760-supplementary.pdf]

Supplementary Information for

Efficient degradation of ofloxacin by magnetic $CuFe_2O_4$ coupled PMS system: Optimization, degradation pathways and toxicity evaluation

Chuanhong Xing, Kang Chen, Limin Hu * and Lanhua Liu *

School of Ecology and Environment, Zhengzhou University, Zhengzhou 450001, China;

chxing@zzu.edu.cn (C.X.); ck915543328@163.com (K.C.)

* Correspondence: liminhu@zzu.edu.cn (L.H.); lhliu@zzu.edu.cn (L.L.)

Summary

Number of Pages: Page S1–Page S12

Number of Texts: Text S1–Text S3

Number of Figures: Fig. S1–Fig. S4

Number of Tables: Table S1–Table S2

This supporting information contains the following Texts, Figures and Tables:

Text:

Text S1. materials and reagents.

Text S2. the characterizations of $CuFe_2O_4$**.**

Text S3. the evaluation of the catalytic performance.

Figure:

Fig. S1. the degradation rate of OFL for various samples ($[pH]_0$=6.5(unadjusted), $[OFL]_0$=10mg/L, $[catalyst]_0$=0.5g/L, $[PMS]_0$ = 0.4mM).

Fig. S2. the EIS curves for $CuFe_2O_4$, Fe_2O_3, and CuO.

Fig. S3. the pH change tendencies for the different initial pH values.

Fig. S4. the first-order kinetics constants for different inorganic anions.

Fig. S5. the effect of radical scavengers on OFL degradation efficiency in the $CuFe_2O_4$/PMS system. Reaction conditions:(pH unadjusted, $[OFL]_0$=10 mg/L, $[CuFe_2O_4]_0$= 0.66 g/L, $[PMS]_0$ = 0.38 mM).

Table:

Table S1. the experimental design matrix generated by BBD

Table S2. the proposed intermediates of OFL degradation over $CuFe_2O_4$/PMS system.

Text S1. materials and reagents.

All the chemicals utilized were of analytical grade AR and were not further purified. The preparation of solutions was conducted using ultrapure water generated by the purification system. Copper nitrate ($Cu(NO_3)_2$·$3H_2O$), citric acid ($C_6H_8O_7$), sodium hydroxide (NaOH), nitric acid (HNO_3), and tert-butanol ($C_4H_{10}O$) were purchased from Tianjin Kemiou Chemical Reagents Co., Ltd. Ofloxacin, iron nitrate ($Fe(NO_3)_3$·$9H_2O$), peroxymonosulfate (PMS, $KHSO_5$·$0.5KHSO_4$·$0.5K_2SO_4$), L-ascorbic acid (L-asc), and L-histidine (L-his) were obtained from Shanghai Aladdin Bio-Chem Technology Co., Ltd. Methanol (CH_4O) and isopropanol (C_3H_8O) were purchased from Shandong Yuwang Industrial Co., Ltd. Chemical Branch. Anhydrous sodium carbonate (Na_2CO_3), sodium sulfate (Na_2SO_4), sodium nitrate ($NaNO_3$), sodium chloride (NaCl), and disodium hydrogen phosphate (Na_2HPO_4) were procured from China National Pharmaceutical Group Chemical Reagent Co., Ltd.

Text S2. the characterizations of $CuFe_2O_4$

The crystal structures of $CuFe_2O_4$, CuO and Fe_2O_3 were characterized on a Bruker X-ray diffractometer D8 ADVANCE with Cu Kα1 irradiation. The morphology of sample was observed through a scanning electron microscope (SEM, ZEISS EVO2.0). Elemental compositions and chemical states of sample were detected by the X-ray photoelectron spectroscopy (XPS, AXIS SUPRA). The specific surface area of catalyst was measured with a Brunauer-Emmett-Teller instrument (BET, with the fully automated specific surface and Aperture analyzer ASAP2460 from Mack Instruments). The total organic carbon (TOC) determination was performed on a TOC-2000 analyzer. The electrochemical performance of $CuFe_2O_4$ was characterized using a CHI660C electrochemical workstation equipped with a standard three-electrode system. The three-electrode system consisted of a saturated Ag/AgCl reference electrode, a platinum wire counter electrode, and an FTO glass-coated $CuFe_2O_4$ sample as the working electrode. Electrochemical impedance spectroscopy (EIS) was conducted to analyze the electrochemical properties, with Na_2SO_4 (0.5 M) used as the electrolyte. The electron spin resonance (ESR) was measured using a Bruker A300 spectrometer.

Text S3. the evaluation of the catalytic performance

The performance of $CuFe_2O_4$ for PMS activation was evaluated with the degradation efficiency of OFL. The degradation experiments were conducted at room temperature in 100 mL glass beakers. All experiments were repeated three times, and the results were expressed as the average values. Typically, a certain dosage of the prepared sample was added to 100 mL of 10 mg/L OFL solution. Prior to add PMS, the suspension was mechanical stirred continuously for 30 min to achieve adsorption-desorption equilibrium. During the process, a certain volume of reactant suspension was withdrawn at specific time intervals and filtered with 0.22 μm mixed cellulose ester (MCE) membrane into sampler vial, followed by storing at 4°C in the refrigerator to stop the reaction before further analysis.

The concentration of OFL was detected by high performance liquid chromatography (HPLC, Agilent 6546, USA) with a diode array detector (DAD). A C18 column was used for detection, and the injection volume was 20 μL. The mobile phase was acetonitrile and water with 0.1% formic acid, with the ratio of 15/85. The flow rate was 1.0 mL/min and the detection wavelength was 288 nm.

The intermediates from OFL degradation in $CuFe_2O_4$/PMS system were identified with an HPLC coupled to a time-of-flight mass spectrometer equipped with electrospray ionization and quadrupole detection (HPLC-ESI-QTOF-MS/MS, Agilent 6546, USA). The mass spectrometer was operated in

positive ion mode with a scanning range of m/z 200-500.

The possible active species were identified with quenching experiments (TBA, MeOH, L-his, L-asc). The molar ratio of quenching agent to PMS set as 100/1 to ensure the quantity of quenching agent was sufficient. The procedures of quenching experiment were same with the degradation experiments but the quenching agents was added into the solution prior to the reaction.

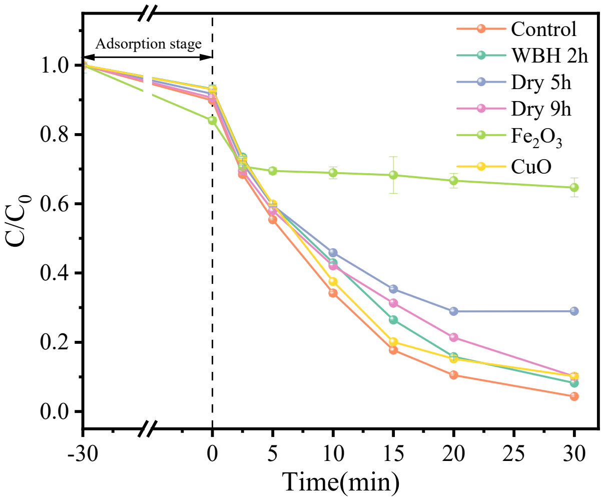

Fig. S1 the degradation rate of OFL for various sample ($[pH]_0$=6.5(unadjusted), $[OFL]_0$=10mg/L, $[catalyst]_0$=0.5g/L, $[PMS]_0$ = 0.4mM).

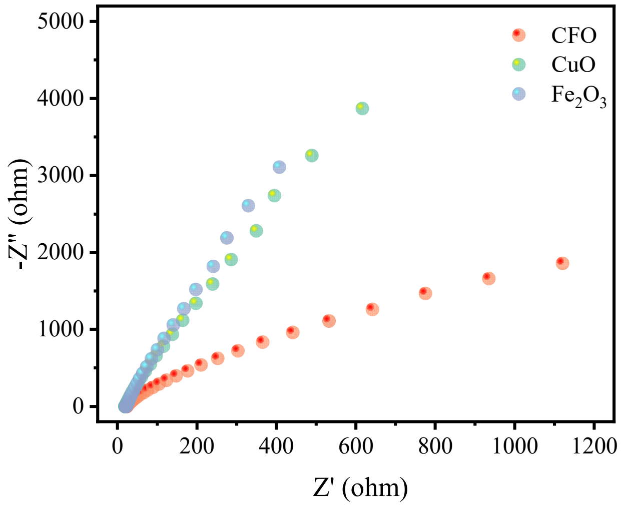

Fig. S2 the EIS curves for $CuFe_2O_4$, Fe_2O_3, and CuO.

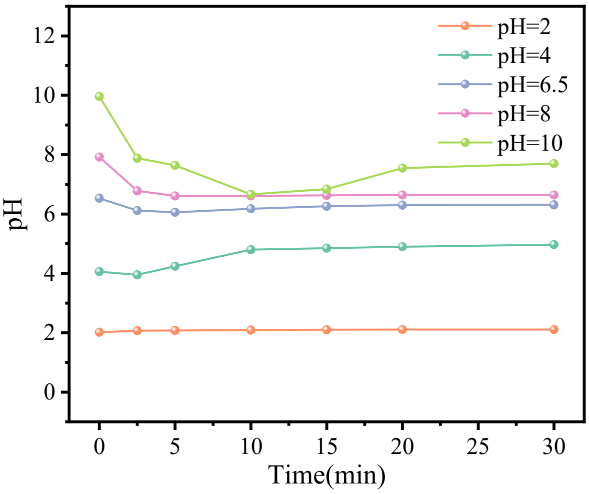

Fig. S3 the pH change tendencies for the different initial pH values.

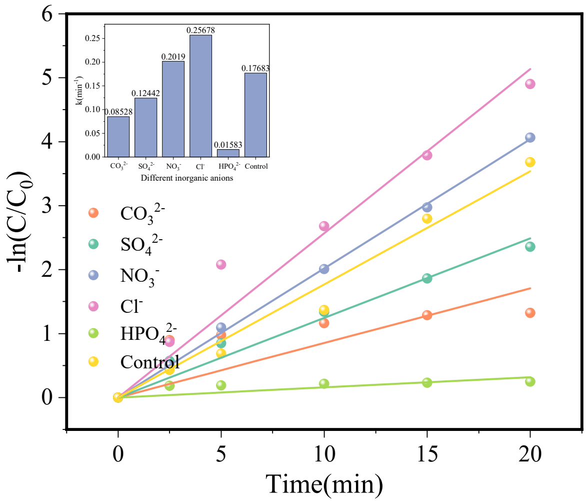

Fig. S4 the first-order kinetics constants for different inorganic anions.

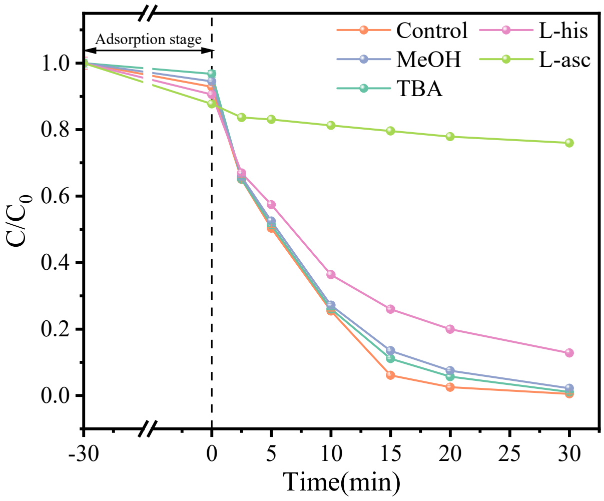

Fig. S5 the effect of radical scavengers on OFL degradation efficiency in the $CuFe_2O_4$/PMS system. Reaction conditions:(pH unadjusted, $[OFL]_0$=10 mg/L, $[CuFe_2O_4]_0$= 0.66 g/L, $[PMS]_0$ = 0.38 mM)

Table S1. the experimental design matrix generated by BBD

Run order	Actual variables			Removal efficiency (%)	
	A	B	C	Experimental	Predicted
1	0.1	0.1	6	34.22	34.89
2	0.7	0.1	6	72.83	67.24
3	0.1	0.4	6	33.62	39.21
4	0.7	0.4	6	99.36	97.55
5	0.1	0.25	2	8.65	3.45
6	0.7	0.25	2	42.08	44.28
7	0.1	0.25	10	38.16	35.96
8	0.7	0.25	10	79.46	84.66
9	0.4	0.1	2	23.58	26.97
10	0.4	0.4	2	26.31	25.92
11	0.4	0.1	10	45.22	45.61
12	0.4	0.4	10	83.55	80.16
13	0.4	0.25	6	90.45	90.43
14	0.4	0.25	6	94.74	90.43
15	0.4	0.25	6	87.45	90.43
16	0.4	0.25	6	91.41	90.43
17	0.4	0.25	6	88.12	90.43

A: catalyst concentration (g/L); B: PMS (mM); C: pH

Table S2. the proposed intermediates of OFL degradation over $CuFe_2O_4$/PMS system.

Compounds	Formula	m/z	Proposed structure
OFL	$C_{18}H_{20}FN_3O_4$	362	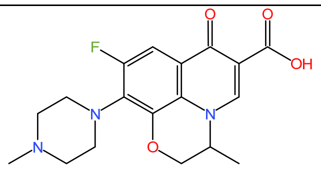

P1	$C_{17}H_{18}FN_3O_4$	348	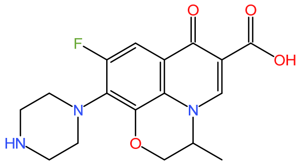
P2	$C_{13}H_{11}FN_2O_4$	279	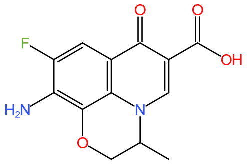
P3	$C_{17}H_{18}FN_3O_5$	364	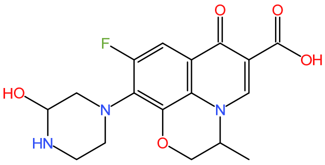
P4	$C_{12}H_{11}FN_2O_2$	234	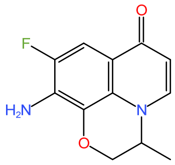
P5	$C_{11}H_9FN_2O_2$	221	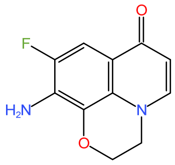
P6	$C_{13}H_{10}FNO_4$	263	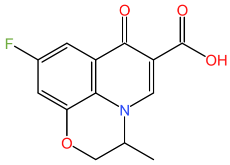
P7	$C_{10}H_6FNO_3$	207	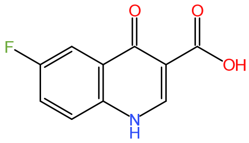
P8	C_9H_6FNO	164	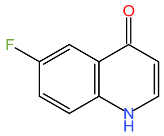
P9	$C_{16}H_{16}FN_3O_6$	366	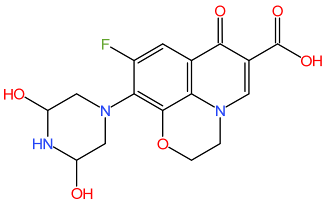
P10	$C_{15}H_{16}FN_3O_4$	322	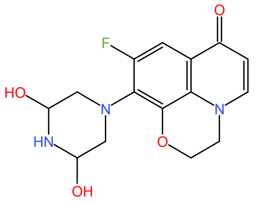

P11	$C_{17}H_{16}FN_3O_4$	346	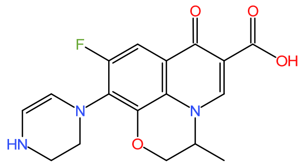
P12	$C_{15}H_{13}FN_2O_4$	305	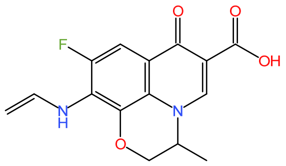
P13	$C_5H_{12}NO$	102	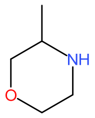
P14	$C_6H_{13}NO$	116	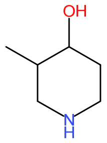
P15	$C_5H_5NO_2$	111	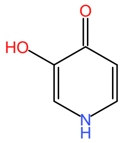
